# Supplementary material for: PCR artifact in testing for homologous recombination in genomic editing in zebrafish
Source: PLoS One. 2017 Mar 31;12(3):e0172802. doi: 10.1371/journal.pone.0172802 (PMC5375128; doi:10.1371/journal.pone.0172802)
Supplement: S1 Fig — Lower case bolded sequence in the 5’ region indicates exon sequence, with red letters indicating the start codon, followed by the ecdysone receptor coding region highlighted in green, the zebrafish codon optimized V2A sequence highlighted in pink and nuclearCFP coding sequence highlighted in blue. The V2A ad CFP plasmids were kindly provided by Harold Burgess [21]. On the 3’ side, grey highlight indicates genomic sequence in the left homology arm. Lower case letters again show exon sequence, yellow underline indicating the SV40 PA terminator sequence in the insert DNA. (PDF) [file pone.0172802.s001.pdf]

AACTCCAGTTCAAGTTCTCTCAAAACTACAATTGCCTCATAATTTATCTCCCTCATATTTCCA
TTACTTTTTTCCTTGATCTAAGGAAATATTAAAGGAATAGATCGTGAAATATTAGAAAAAATG
CTGAATTAATTTACTCAATGCATAAATCGCAACATAAATCTTCTTTACTATTCTACTAAATAAA
AAGCCAACTACATCTTAGATGGCCTTAGAGTAAGTTAACAGCAAATGTTCATTTTTAGAAAA
AAGTTCTGAATAAATGGTGATGGTGAGTAAACACAAAGCCAAGGCAAGTTTATTTATAAAGC
ACATTTATTACACAGTGGCAACTCAAAGTGCTTTACATGAACAGGAATAAAAGAGACAAGTA
TAAGAAAATATAAACAAACAATAACAGTGATTAAAAACAGATTAAAATGTGTTAAAACAGGTC
TTAAAAGAATGAAAAAGAAAAGAAAGACATAATGTAAGCCGTATTCCTTTAAATCTACACAG
TATCAAAATCAGAAACAGCCTTCGGAGGAAAGATTTTAATTATAACATAGTAGGTTTTTGATT
AATATTTATATTTAATGACTCAGAGGGCTTCCGCTCTGGTTTTTATGAACAAGTGCTCTCCA
TTGGAATCTTACACAGCATCTGTTTTTCGTCGTCTTCTCTTGCCCCTCCTTTTCTCACTTTCA
CATGAAAACCTCTTGACTCGGTGCTCCCTGCCTCAATCCTGGATAATTCTCTCTCTGCGGG
ATTGAAAGATTTCTCTAGGCGAGAGAGCATGAGATTACACTGCCAGATTAATGTGCAGGAG
TAAACCCACACTGCTCTGCAACATAATCTCATCCTGGCACACACAAACACTGTCTCTCATTC
ACAATAGTTACCTCACTGTTTCCCTGCCACCCTTCAGagtgatgtcactgtgcctgccagggggtggtcgtcc
cctgccggagtcgcacagcctctgacgcccgcgggatggactgagtgagtgtagccaaagccgagagcaatgaagctactgtcttct
atcgaacaagcatgcgatatttgccgacttaaaaagctcaagtgctccaaagaaaaaccgaagtgcgccaagtgtctgaagaaca
actgggagtgtcgctactctcccaaaaccaaaaggtctccgctgactagggcacatctgacagaagtggaatcaaggctagaaaga
ctggaacagctatttctactgatttttcctcgagaagaccttgacatgattttgaaaatggattctttacaggatataaaagcattgttaacag
gattatttgtacaagataatgtgaataaagatgccgtcacagatagattggcttcagtggagactgatatgcctctaacattgagacagc
atagaataagtgcgacatcatcatcggaagagagtagtaacaaaggtcaaagacagttgactgtagaattcctggatatggccgact
tcgagtttgagcagatgtttaccgatgcccttggaattgacgagtacggtgggctgcagatgaggcctgaatgtgtcatacaggagccc
agtaaaaataaagacaggcaaagacaaaagaaagacaaaggaatattattacctgttagtacgaccacagtcgaagaccacatg
cccccgatcatgcaatgtgatccacctccgcccgaggccgccaggattcacgaagtcgtcccgaggtatctttcggagaagctgatg
gagcagaacaggcagaagaacataccaccattgtcggcgaatcagaagtctctgatcgcgaggctcgtgtggtaccaggagggat
atgagcagccctccgacgaggatctcaaaagagtaacgcagacttggcagtcggatgaagaggacgaggaatccgatctaccctt
ccgccagatcacggagatgacgatcttaacggtccagttgatcgtcgagttcgccaagggtctaccgggcttttcgaagatatcacagt
ctgatcaaatcaccttattaaaagcctcgtccagcgaggtgatgatgctgcgggtggcgaggcgatacgacgccgcgtccgacagc
gtgctgttcgccaacaacaaggcgtacacgcgcgacaactaccgccaaggcggcatggcctacgtcatcgaagacctcctacactt
ctgccggtgcatgttcgcgatgggcatggacaatgtgcactttgcactgctcacggccatcgttatattctcagatcggcccgggctcga
gcagccgtcgctggtagaagagatccagagatactacctgaacacgttgcgaatttacatcatcaaccagaacagcgcgtcgtcgc
gctgcgccgtgatctacggcaggatcctgagcgtgctgaccgagctacgcacgctcggcacgcaaaactccaacatgtgcatctcgc
tgaagctgaagaacaggaagctgccgccgttcctcgaggagatctgggacgtggcggaggtggccacgacgcatcccacggtgct
gccgcccaccaacccggtggtgctaggaagcggagagggaagaggaagcctgctgacatgtggagatgtggaggagaaccctg
gacctgctcctggaagcatggcaggtggaaaagcaggtaaagacagtggcaaagccaaggcgaaagcagtgtctcgctcgcaa
agagctggtcttcagtttccagtgggacgaatccacaggcacttgaagacacgcactacaagccatggtcgagtaggagcaacagc
agccgtttacagcgcagccattcttgaatatctcacagctgaagttttggagttggcaggaaatgcttcaaaagacctgaaggtgaagc
gcatcactcctcgacatttacagctggccattcgaggagatgaggagctcgattcccttatcaaggccactattgctggaggaggtgtg
attccacacatccacaagtctctgattggtaagaagggccagcagaaaaccgcaatggtgtcaaaaggagaggaactgtttactgg
agtcgtgcctatcctggtggagctggatggagatgtgaatggacacaagttctctgtgtctggagagggagagggagatgctacatat
ggaaagctgacactgaagttcatctgtacaacaggaaagctgcctgtgccttggcctacactggtgacaacactgacatggggagtg
cagtgttttgctagataccctgaccacatgaagcagcatgacttcttcaagtctgctatgcctgagggatatgtgcaggagagaacaatc
ttcttcaaggatgatggaaactacaagacaagagctgaggtgaagtttgagggagacacactggtgaacagaattgagctgaaggg

aattgacttcaaggaggatggaaacatcctgggacacaagctggagtacaatgctatctctgacaatgtgtacatcacagctgacaa
gcagaagaatggaatcaaggctaacttcaagatcagacacaacattgaggatggatctgtgcagctggctgaccactaccagcag
aacacacctattggagatggacctgtgctgctgcctgacaaccactacctgagcacacagtctgctctgagcaaggaccctaatgag
aagagagaccacatggtgctgctggagtttgtgacagctgctggaatcacactgggaatggatgagctgtacaag**tga**actatagtg
agtcgtattacgtagatccagacatgataagatacattgatgagtttggacaaaccacaactagaatgcagtgaaaaaaatgctttattt
gtgaaatttgtgatgctattgctttatttgtaaccattataagctgcaataaacaagttaacaacaacaattgcattcattttatgtttcaggttc
agggggaggtgtgggaggttttttaattcgcactcaaccaggacaccatgccacacccсagacaacactcatatctgtgtcttctaatg
gtcctatgtgttctactctctgccacggaggcaccatactgtcaaagccagagtctcgaaaaaaaaacaacaacaacgaatgcttta
cttgatgctgaacaaaccaggccttagaaaacctgccacgcccatgaggcttttccccctctggagagcagccttaaggtctcccctca
ccatgccgatcagcagtccactctaccggaggttttatattcatgttatctctaaatgtgatctacgtttgtgaatgtttgtgaaaaagagag
acagaacaaacagtgtatgtatgcgtgcaagtatgaatggatgaatgtgcacagctgtttaataaatctttcccagtgaggacaCCA
GAGTTAAGCCTCAGTTGATGTGTAAAAATGATTCCAAATACACTTATAGTCCGATTTGATCC
GACTCTTTTTTACTATACGTTTAGTAATCTACTAGTAGATTACTCTCTAATGACAAGTCAGAT
TTTAAAACATTTCAACTCTCTAGAGATAGAAATATAGGATAGATACATCAGCTTTTTTCAAAA
TCTTTGAAACGATTAGCAGAAAGCTCTCTTAGAGGATTTTAGCCTCTATCTTTGGGCAAGTG
TTCAGGGTTTGGCTGATTAAGCGTGTCCCTGTTAGCTCCTGCTGGTAAATGCCATAGCTAC
ACCATCTGGCTGGAAAAAAGGAAGGTAACCTAAAAAAAAGGTGTCAGTACATAGAATAAAG
GCTTTTGCATGACACTTTTAGTAATAAAAGCGCAGACTATTTAAAACTGTTTTTAAAGCTCAA
GTGTTTGAAAATAAAACTTCCCTGTGTTAATTATTAAAAAATGTGATCATGAAAATTGTGAGA
TGTAAAACATATCCTAAATTCTATATATCTATATAACACAAACTTCCACAGCATATCAAATGT
AGCATTTCCATATGCGCTG

S1 Fig. Sequence of the donor DNA construct. Lower case bolded sequence in the 5’ region indicates exon sequence, with red letters indicating the start codon, followed by the ecdysone receptor coding region highlighted in green, the zebrafish codon optimized V2A sequence highlighted in pink and nuclearCFP coding sequence highlighted in blue. The V2A ad CFP plasmids were kindly provided by Harold Burgess [21]. On the 3’ side, grey highlight indicates genomic sequence in the left homology arm. Lower case letters again show exon sequence, yellow underline indicating the SV40 PA terminator sequence in the insert DNA.
