# Supplementary material for: PCR artifact in testing for homologous recombination in genomic editing in zebrafish
Source: PLoS One. 2017 Mar 31;12(3):e0172802. doi: 10.1371/journal.pone.0172802 (PMC5375128; doi:10.1371/journal.pone.0172802)
Supplement: S2 Fig — Primers used for cloning are shown. (PDF) [file pone.0172802.s002.pdf]

Probe #1 seq (660bp)
CTTGCGTCTAAGAACAAGCCAAAACTAGTAATCAAATTAACAGAAATAACTGACTACAGATG
TAAAGTCTGTCTAATCTGTCTAGTTTTGGGCTATTCTTAGATGTCTTTTAGATTTTTACTGAC
AGCCCAAGTTCAGCCTTGTTTTAGCCAAACTGTCTTAGTTTAGATGTCTATTAGACGTCTAT
TAAACACAAAATTGTTTGCTGGGCATGGACTCCATTAGGCATGAGACGATAACCGTTTTCAA
GGTATACCGAGGTTTGGAAAAGTCAAGGTTTTAAAACCGCCAAAATTTTCTGTTATAATCAT
TCCTAAGGTGTGTAAGATTTTTTTATTTACATTTTTTTAGGACTACAGTATCTCCAGCAGAGA
ATATATCAAAAGATGCCATTTTAAATTGTAAAGGAATCTGTGTTTTTGAAACTAATAAAGACA
GCAGAAGTCAATGATTCATTTAAATTCTTTAGCCTGACATGTTTACTGCTCCAAAATACTTTA
CATGTTTCTCAAAATAAAATATATTTTGTTTAAAGGGGAAAAAATGATTGTTTTATTTTAGAG
CAGTAATCACAATACTGTGAAACCGTGATATTTGTTTCCAAGGTTATCATACCGTCAGAATT
TATACCATAATACTACTGAGAAAAGGAACCACCAT

We used primer pair (F-CTTGCGTCTAAGAACAAGCCA, R-ATGGTGGTTCCTTTTCTCAGTAGT) for template DNA cloning.

Probe #2 seq (791bp)
GCTTTCCCTGTAGCCGAGATGTTTACAGATCAGCCGTTAGTTGTATTGTTTTGTCGTGTCAG
TTCAGCTGAACAAACATTTAATTCTTTATCCGCCAGGCAGCCAGATAAGAGGCGAATGTGC
CATTTTGCCAGTGAGAACACAGACAACTGTGACAGTCAGATAAAGATACAAGGATACAACA
AGTGAGTGGCCTTGAACAGCACTTGCGGTCAATATCTAAACACTCCCTTGTGATTAAAAGT
CTGAGGGGAATGAACATAGGATGGAAACACTAATGGCAGCCGCCCAATGACCATTAAGCA
TCCCAGCCTTTATGTAATGAAGTCCCACATCTTCGCTATCAAGGCACGAGCTGCTTCTTTTG
GTTCAGACTGTCCACATTAAGACATTTACAGTGTGAGCATGGGTATGTGTGTGTGTGATGA
GACGTTGACAGGGCTTTGTAGTGGGAACAGAGGAGCCTTGTGTCCTCATGTTTTATTCATG
TCCAGCTGCGAGATAAACACAGAGGCAGGTAGTCACGGAGATGCCGTCCACAGAGCAGG
CGTGAGGCCGGTCATGGACGCACAGCTGTCGGATAACGTGCATATAGACGATAAGTGTCG
TTATTGTTGGCCCAATAGACTGTCCCCTGCTGTTTGGTGGCATTGAATAGGCAATTTTCAAG
GAATAGTTCAGGAGTTTTCTCTTTCTAGAAGCCACCTAGTAAGCATTTTTTTTTAAAAAGATG
TCTAAAAGACGTCTAAACATAGTCGTCTTGGCTAAAACAAGGCTACATTTGGACTGTC

We used primer pair (F-GCTTTCCCTGTAGCCGAGATG, R-GACAGTCCAAATGTAGCCTTG) for template DNA cloning.

Probe #3 (707bp)
TCTTCCCTCTCCGCTTCCTAGCACCACCGGGTTGGTGGGCGGCAGCACCGTGGGATGCG
TCGTGGCCACCTCCGCCACGTCCCAGATCTCCTCGAGGAACGGCGGCAGCTTCCTGTTCT
TCAGCTTCAGCGAGATGCACATGTTGGAGTTTTGCGTGCCGAGCGTGCGTAGCTCGGTCA
GCACGCTCAGGATCCTGCCGTAGATCACGGCGCAGCGCGACGACGCGCTGTTCTGGTTG
ATGATGTAAATTCGCAACGTGTTCAGGTAGTATCTCTGGATCTCTTCTACCAGCGACGGCT
GCTCGAGCCCGGGCCGATCTGAGAATATAACGATGGCCGTGAGCAGTGCAAAGTGCACAT
TGTCCATGCCCATCGCGAACATGCACCGGCAGAAGTGTAGGAGGTCTTCGATGACCTCGG
CCATGCCCGCCTTGCGGTAGTTGTCGCGCGTGTACGCCTTGTTGTTGGCGAACAGAATGC
TGTCGGACGCGGCGTCGTATCGCCTCGCCACCCGCAGCATCATCACCTCGCTGGACGAG

GCTTTTAATAAGGTGATTTGATCAGACTGTGATATCTTCGAAAAGCCCGGTAGACCCTTGG
CGAACTCGACGATCAACTGGACCGTTAAGATCGTCATCTCCGTGATCTGGCGGAAGGGTA
GATCGGATTCCTCGTCCTCTTCATCCGACT GCCAAGTCTGCGTTACTC

We used primer pair (F-TCTTCCCTCTCCGCTTCCTA, R-GAGTAACGCAGACTTGGCAGT) for template DNA cloning.

Probe #4 (747bp)
GTAGCATTTCCATATGCGCTGAAAGAATGAGGTATTTTTGCACACACTGTATAGTTGAAAGA
AGGTCTGTGCTTTAAACATGTTCAAAATGTTGTCGTTTTATATCAAAATCCATTGTTTTATTTA
TTTTTTTATTTGTACTGGGGGGTGGGGGTAAAGCTAAAAGTTTGCATTATCTTGGTTCCTTT
TTTTTCAATTTTAGATTGCAAAACATGTCAGCATAGACATTAAATATAATTTCACCTAACAGG
TAAAACTCAACTACTCATTCACAAAGTTGTCAATAGGGCTGCATAATAATGGAAAAATCTGA
TATTGCAATATTGAATAAATATTAAATTTGCGATAAATATTGCGATATATATGGTTTCACAAG
ATGTTGGAATAGCACTATTTTTCTAGTTTTCTTGGAAGTCTAACACTATTCAGGTACAGGAAT
TAAATAACCACAATTTAAAAGAAAGTTCTTACTTTGCCTTGACTTGTCTCTAGTCCAAATAAC
TAAAAAAAAATCTTAGATCAAGTAAAAATATAAAAAAATAAGACAAAATTAAGATTCCCCCCC
CCCCCCCTTAAAACATGCAAATTTATCTCCCAGTGGAGTAAAATAATTTAGTTTTTACTTTGA
AATGTAGATATTTGGACTTAAATCAAGACAAAGTTTAAGGTGCGAAAAACTTTTTTTTTTCAT
AAATTATATAGAATTCCATAAGAAAAAAAATTAAATACAATTCTGTGGCTCTTGGG

We used primer pair (F-GTAGCATTTCCATATGCGCTG, R-CCCAAGAGCCACAGAATTG) for template DNA cloning.

S2 Fig. Sequences of Southern blot probes. Primers used for cloning are shown.
