# Supplementary material for: PCR artifact in testing for homologous recombination in genomic editing in zebrafish
Source: PLoS One. 2017 Mar 31;12(3):e0172802. doi: 10.1371/journal.pone.0172802 (PMC5375128; doi:10.1371/journal.pone.0172802)
Supplement: S3 Fig — Restriction sites shown in bold. (PDF) [file pone.0172802.s003.pdf]

Primer pair: F- GAT**CTCGAG**GTTGCCAAAGAGACAGATATG,
R- GAT**TCTAGA**TTAAATCAGGTAGTCAATCTC.

CTCGAGGTTGCCAAAGAGACAGATATGTTTGAAGAGGTTGAGTTTTGTGTGATAAATGGCG
ATGATGAACATTCCAAAGCAGAGCTTGAAAAAGGGGTGGCGCGCTGTGGAGGCATCGTTG
TGCAGAATCCTGGAAAGGACACGTACTGCGCCATTGCAGCTGTGCAGAACATGAGAGTGA
AGAACCTCATCTCATCAGATCAGCATGATGTGGTGTGGGCTACTTGGCTTTTAGAGTGTCT
GGAAAATAAGCAGGTTATCCCATGGCAACCACGTCACATGATCCACATGTCACCTTCCACA
AGGGAACACTTCGCTAAAGAGTATGATCAGTATGGAGACAGTTATTACGTTGACACTAGTG
AACAGCAGCTGAGGGATGTCATTGAAAGGATCAGCTCCGCAGAAGTCAAGAGTCTGTCTG
TGGCTCAGATAGAAGCAGAGAATGCTTGGGATGACTTGCCTACTAGCATTTTTAGGCCTTA
CAGTGCATACTTTGACCGGTGTGCTGACATAGGAGATCCCAAGTCTGTTATCTGCGGTACG
TGTTTAGACACTCGTGCCTTGGAATTCAGATTTCATGGCGGTAAAGTTGTGGAGAAACTCA
AAGAAGGAATCTCACATGTGGTAGTAGAAGATATGAAAAGGGCTCTGGACCTAAAGACACT
GAGGCGACTTCATGCCAAGAAGTTTAAAATTGTTCATGAATCATGGGTTACTGATTCCATCA
AAGCAGGCCATGTTAAGGATGAGATTGACTACCTGATTTAA**TCTAGA**

S3 Fig. Primers used to isolate zebrafish dominant-negative Lig4, and sequence of the cloned product. Restriction sites shown in bold.
