# Supplementary material for: PCR artifact in testing for homologous recombination in genomic editing in zebrafish
Source: PLoS One. 2017 Mar 31;12(3):e0172802. doi: 10.1371/journal.pone.0172802 (PMC5375128; doi:10.1371/journal.pone.0172802)
Supplement: S4 Fig — Restriction sites shown in bold. (PDF) [file pone.0172802.s004.pdf]

Primer pair (F- GAT**CTCGAG**ATGACTATGCAGTGTGTGCGCAGTGTG, R-
GATTCTAGATCATTTAGAGAGCTGCATGGTCAGGGC).

CTCGAGATGACTATGCAGTGTGTGCGCAGTGTGTACACTAGTAAGATCATAAGCAGCGAC
AGAGACCTGGTTGCTTTGGTGTTTTATGGGACTGAGCAGAGCAAGAACCCCAGAAACTCCT
TCAAGCATGTTTACGTCTACCACGACTTGGAGTCCCCTGGAGCAAAACGTGTACAGGATAT
TGATAAACTGAAAGGTGATAAAGGTGGTCAGTTTGCTGAAAAGACCATGGGCAGTGGGGA
AACTTCTCTCGGAGAGGCTCTGTGGTGCTGTTCCAACCTTTACAGTGACATCAAGTTGCGA
CTGTCACACAAGCGCCTCATGATATTCACATGCAGGGATGAGCCTCATGGAGGAGACAGT
GCAAAAGACAGACAGGCTCGTACAAAGGCTGCTGACCTAAAAGAGACAGGTGTAGCCATT
GACTTGATGCACCTCTCAAAGCCTGGAGGGTTTGATGTTTCACTGTTCTTTTGCGATATTGT
AAGTCCTCCAGAAGATGAAAGTGATCTCGGCCTTCAGATTGAGCCCTGCAGGAAACTGGA
GGACCTGCAGAAGAGAGTCAGAGCAAAAGAGTTAAAGAAAAGAGCTCAATGCAGGTTAAC
GTTCTCCCTTGGAGAAGGTGTCGATCTGGCTGTTGGCGTATACGTGTTGGCCAGGACTGC
TATGAAGCCTTCTGCTGTCAAACTTTACAGAGATAATAATGAACCTGTACGGACTAAATCCC
GTCTCTTCCACACTCAAACTGGTGGCATTTTATTACCAAATGATACAAAGAGGGCACAGGTT
TATGGGCAGAAGCAGATTGTGATGGAAAAGGATGAGGTGGATGAGATAAAGAAGTTTGAT
GATCCAGGATTGGTACTGATTGGATTTAAGCCTATAGATCGCCTCAAACTGCACCATCATTT
ACGACCTGCTCTTTTCATATACCCTGAGGAGGAGCAGATATCAGGAAGCTCCTGTATGTTT
ACAGCTCTGCTTTTGAAGTGCTGTGAAAAAAATGTATTTGCATTGTGCAAATACATCCCTCG
TCGTAACACTCCACCTCGGTTTGTGGCACTAGTGCCCCAGAGAGAAGAACTGGATCAGAG
TCAAACCCAGGCAACACCTCCAGGCTTCCATGTGATTTATCTTCCCTTTGCTGATGACATAC
GCACTGTGGACCCTCACGTTGGTCCCACAGCCTCGGATGAGCAGGTAGACAAAATGAAAG
AGATCGTGCACAAGCTCCGCTTTAAATACAGGAGTGATGCATTTGAAAACCCAGTGCTGCA
ACAGCACTACAGTAATCTGGAGGCTTTGGCTCTGGATATGCTTTCACCTGAACCCATTGAG
GATTTGACAATGCCAAAGGTAAAAATGATGGACGATCGGCTTGGTCCGCTGGTTCAAGAGT
TCAAAGATTTGGTCTATCCTCCTGATTATAACCCAGAAGGGAAACCTGCAGCTAAACGCAA
ACCTGCTGAATCTGGTGGTGGTGCTGCTGAAAAGAAGGCCAAAGTTGAGATTTCCGAAGA
AGAGCTGAAAATTCATGTCGCAAAAGGCACTTTGGGAAAACTGACAGTGCCTGTACTAAAA
GATGCATGCAAACAGTTTAATATACGTACAACAGGAACCAAGAAACAGGAGCTTATCGATG
CCCTGACCATGCAGCTCTCTAAATGA**CTCGAG**

S4 Fig. Primers used to isolate zebrafish dominant-negative Ku70, and sequence of the cloned product. Restriction sites shown in bold.
